# Artificial Incubation for Infants

**Published:** 1883-05

**Authors:** 


Artificial Incubation for Infants.—Dr. Tavernier, physician to a foundling hospital at Paris, has tried an experiment with a view to lessen the enormous mortality among the infants under his care. A prematurely-born infant of miserable physique, was made the subject of the experiment. It was placed in an incubator, made on the model of the artificial incubators for chickens. It was a box covered with a glass slide, furnished with a soft woollen bed, and kept at a temperature of 86° by suitable means. The child was placed in this and left in the dark with a nursing-bottle. On the second day it ceased to cry and sank into a deep sleep, which continued during the 60 days it remained in the incubator, the only wakeful intervals being when it was taking nourishment. At the end of the time it was as well grown and strong as a child a year old. Another experiment was tried, and was equally successful. The system was thus applied with all convenient speed to the 360 infants in the hospital. Their average weight was then 16 lbs.; average age, eight months, three days. Only one died from congenital hydrocephalus, another was reclaimed. The rest remained in the incubator for six months. The average weight was then 24 lbs. An ordinary observer would have said that the youngest was at least three years old. All learned to walk within a week after leaving the incubator, and most have since learned to talk.—*Journal of Chemistry.*

